# Differences in physician opinions about controversial issues surrounding contralateral prophylactic mastectomy (CPM): A survey of physicians from accredited breast centers in the United States

**DOI:** 10.1002/cam4.2914

**Published:** 2020-03-11

**Authors:** Katharine Yao, Richard Bleicher, Meena Moran, Cecilia Chang, Jill Dietz, Vered Stearns, James Connolly, Terry Sarantou, Scott Kurtzman, National Accreditation Program for Breast Centers Data Working Group

**Affiliations:** ^1^ Department of Surgery NorthShore University Healthsystem Evanston IL USA; ^2^ Department of Surgery Fox Chase Cancer Center Philadelphia PA USA; ^3^ Department of Radiation Oncology Yale Medicine New Haven CT USA; ^4^ Biostatistical Core NorthShore University HealthSystem Research Institute Evanston IL USA; ^5^ Department of Surgery University Hospitals Cleveland OH USA; ^6^ Department of Oncology Johns Hopkins Medicine Baltimore MD USA; ^7^ Department of Pathology Beth Israel Deaconess Medical Center Boston MA USA; ^8^ Department of Surgery Carolinas HealthCare System Charlotte NC USA; ^9^ Department of Surgery Waterbury Hospital Waterbury CT USA

**Keywords:** contralateral prophylactic mastectomy, insurance CPM, quality measure CPM

## Abstract

**Background:**

Indications and insurance coverage for contralateral prophylactic mastectomy (CPM) and CPM as a quality measure are controversial. Few studies have examined physician opinions on these issues.

**Methods:**

A cross‐sectional survey of multi‐specialty physicians at the National Accreditation Program for Breast Centers from 2017‐2018 examined opinions on insurance coverage for CPM, CPM as a quality measure, and indications for CPM. A multivariate logistic regression was used to assess physician and facility factors associated with likelihood to recommend CPM.

**Results:**

Of 2412 physicians, 1226 responded from 382 facilities for a physician response rate of 50.8%. There were 300 (24.5%) medical oncologists, 316 (25.8%) radiation oncologists, 248 (20.2%) plastic surgeons, and 322 (26.3%) oncologic or general surgeons. Three hundred and ninety‐eight (37%) physicians favor insurance coverage for all patients and 520 (46.6%) for patients at average CBC risk. Four hundred and fifty (40%) of all physicians felt physician specific rates of CPM should be a hospital quality measure. *BRCA* deleterious mutation carrier status was the most common indication to recommend CPM (n = 1043; 92%) and 684 (60.2%) physicians discourage CPM for average contralateral risk (CBC) patients. After adjusting for physician and facility factors, the only significant predictor of higher likelihood to recommend CPM for average CBC risk patients were plastics surgeons (OR = 8.3 (95%CI 2.4‐29.1)) *P* = .0009).

**Conclusion:**

There is consensus among physicians on the most appropriate indication for CPM but opinions vary on CPM as a quality measure and insurance coverage for CPM. These findings can help guide discussions on CPM among a multidisciplinary team of physicians.

## Graphical abstract

A survey of over 1200 physicians working at accredited breast centers in the United States showed there is consensus among physicians on the most appropriate indication for contralateral prophylactic mastectomy (CPM) but opinions vary on CPM as a quality measure and insurance coverage for CPM.

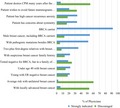

## 1 BACKGROUND

The routine use of contralateral prophylactic mastectomy (CPM) is controversial. There is no consensus on indications for CPM and few national guidelines make outright recommendations for CPM. However, many of the guidelines do discourage CPM for women at average contralateral breast cancer risk (CBC).^1^, ^2^, ^3^ There are few resources to guide physicians on when to recommend a CPM or when to discourage it. Since approximately half of surgeons report some discomfort in performing CPM at some point in their career,^4^ it appears prudent to explore physician opinions on indications for CPM. It is also not clear if other subspecialties would make similar recommendations for CPM as surgeons. Newly diagnosed breast cancer patients often have encounters with a variety of physician subspecialties, particularly plastic surgery, prior to making their definitive surgical decisions. Many newly diagnosed patients are seen in the multidisciplinary clinic setting where they have the opportunity to consult with surgeons, medical and radiation oncologists, and/or plastic surgeons shortly after receiving their breast cancer diagnosis. Only one small study has directly queried plastic surgeons,^5^ who often play a significant role in local surgical decision‐making, about CPM.

Other controversial topics surrounding CPM, including insurance coverage for CPM and whether CPM should be a quality measure have not been previously addressed in other studies. It is not clear how often insurance companies deny coverage for CPM. Lastly, with a renewed focus on quality and value‐based care, many hospitals have focused on more extensive surgical procedures and their complication rates. Studies have shown that CPM with and without reconstruction surgery increases complication rates compared to unilateral mastectomy^6^, ^7^, ^8^ and can also impact timely care.^9^, ^10^ These factors have called into question whether CPM should be a quality measure tracked by hospitals or even medical societies.

To understand physician's recommendations for CPM and their opinions on insurance coverage and whether CPM should be a quality measure, we conducted a cross‐sectional survey of physician specialties from a network of breast centers that have been accredited by the National Accreditation Program for Breast Centers (NAPBC). The NAPBC accredits breast centers that fulfill established standards of care and NAPBC accreditation has been shown to improve performance with certain breast specific quality measures.^11^ The NAPBC is made up of academic and community breast centers across all regions of the United States making our findings generalizable across many types of breast center practices in the United States. The diversity of the centers included in this study enabled us to assess whether opinions on CPM vary by physician demographics or practice factors in a comprehensive fashion across multiple breast centers. These findings provide timely and relevant novel information that has not been previously reported.

## 2 METHODS

### 2.1 Study design and participants

A multidisciplinary, physician‐based survey was designed specifically for breast centers accredited by the National Accreditation Program for Breast Centers (NAPBC). The survey was distributed to NAPBC accredited centers, and if completed, would fulfill one of three annual quality improvement (QI) projects that was required (as per Standard 6.1) for accreditation. In order to have a balanced composition of physician specialties, the instructions stipulated that each NAPBC center had to return the completed survey from one of each physician subspecialties: medical oncology, radiation oncology, surgery (general or surgical oncology), and plastic surgery. Centers that returned completed surveys from a minimum of three of the four specialties were given credit for the QI project and all duplicate surveys were excluded. This study was approved by the Institutional Review Board at the corresponding author's institution.

### 2.2 Response rate

The survey was distributed to the all NAPBC breast centers across the United States that were accredited at the time of initiating this study. Physician response rates were calculated by multiplying the number of centers by the required number of physician surveys per center (4 × number of centers) for the denominator, and total surveys returned minus duplicate surveys for the numerator. Facility response was calculated as the number of centers that participated in the survey divided by the total number of centers the survey was sent to. Duplicated surveys or surveys missing over 50% of responses were discarded.

### 2.3 Development of the survey

Question topics were initially developed by investigators (list initials in parenthesis: (KY, RB, JC, SK)) The University of Chicago Survey Lab, who was then given these topics, conducted conversational interviews with three surgeons, one medical oncologist and one radiation oncologist to develop questions for the survey questionnaire. These interviews helped the Survey Lab determine which indications for CPM were most pertinent, and in order to exclude nonuseful questions and to ensure that the wording and order of the questions was without bias. The survey was then a 34 item ad hoc questionnaire that covers physician opinions and perceptions of insurance coverage for CPM, whether CPM should be a quality measure and indications for CPM (See Appendix S1 for a copy of the survey).

### 2.4 Physician and facility characteristics

Self‐reported data from each physician on specialty, gender, age, years in practice, and number of patients with breast disease seen per week were collected. Location of physician practice was aggregated into Northeast, Midwest, South, and West regions of the country. Affiliation with a medical school was recorded for each facility.

### 2.5 Insurance coverage for CPM

To gauge physician opinions of insurance coverage for CPM, we asked physicians to categorically classify their opinion on indications for coverage (ie, favor coverage, neutral, oppose coverage) for five clinical scenarios and for “all cases” or “no cases.” Physicians were also asked if an insurance company had ever denied coverage for CPM for any of their patients.

### 2.6 CPM as a quality measure

To determine opinions on whether physician specific rates of CPM should be a quality measure, physicians were asked if CPM rates should be added as a quality measure for hospitals and medical societies. Responses were recorded as Yes (definitely/probably yes, definitely/probably no, no opinion).

### 2.7 Indications for CPM

To determine when physicians felt CPM was indicated, we presented reasons for recommending CPM based on patient preference, hereditary factors, and patient age. Physicians were asked if they felt CPM was indicated (strongly indicated, strongly discouraged, neither indicated nor discouraged) for these reasons. Questions are listed in the Supplement.

### 2.8 Statistical analysis

Survey respondents were categorized by self‐reported physician type. Survey responses were summarized using descriptive statistics. Physician opinion on insurance coverage, CPM as a quality measure, and indications for CPM were stratified by physician type and compared using a chi‐square test. Individual multivariable logistic regression models were performed to identify likelihood to recommend CPM for different clinical scenarios adjusting for physician and facility variables. A model confined to plastic and breast surgeons was performed to determine associations between physician and facility variables and recommendation for CPM. Missing data were excluded from analysis. All statistical analyses were performed using SAS 9.4 (SAS Institute Inc, Cary, NC). A value of *P* < .05 was considered statistically significant.

## 3 RESULTS

The survey was distributed to 603 NAPBC centers, of which 382 replied, for a facility response rate of 63.3%. Subtracting 46 duplicate surveys, there were a total of 1226 physicians who completed the survey from these centers, for a physician response rate of 50.8%.

### 3.1 Demographic characteristics of the physicians and facilities

Table 1 shows demographic characteristics of the physicians responding to the survey. The subspecialties comprising the physician cohort consisted of 300 medical oncologists (24.5%), 316 radiation oncologists (25.8%), 248 plastic surgeons (20.2%), and 322 surgeons (26.3%). Their median years in practice were 14 years (range 0‐45 years).

**Table 1:** Demographic factors of the physicians

	N	%
*Physician Characteristics (*N* = 1226)*		
Practice medicine as a		
Medical oncologist	300	24.47
Radiation oncologist	316	25.77
Plastic or reconstructive surgeon	248	20.23
Surgeon	322	26.26
Other	9	0.73
Unknown	31	2.53
Years in Practice, median (range)	14 (0‐45)	
<5	168	13.70
5‐9	212	17.29
10‐15	236	19.25
16‐20	166	13.54
>20	398	32.46
Unknown	46	3.75
Number of patients with breast disease seen per week over the past 3 months		
<10 patients/week	326	26.59
10‐29 patients/week	445	36.30
30‐49 patients/week	207	16.88
>50 patients/week	205	16.72
Unknown	43	3.51
Gender		
Male	639	52.12
Female	543	44.29
Other	3	0.24
Unknown	41	3.34
Age (years), median (range)	49 (31‐80)	
30‐39	202	16.48
40‐49	399	32.54
50‐59	339	27.65
60‐69	188	15.33
>70	21	1.71
Unknown	77	6.28
*Facility Characteristics (*N* = 383)*		
Location		
Northeast	87	22.72
Midwest	121	31.59
South	122	31.85
West	51	13.32
International	1	0.26
Unknown	1	0.26
Affiliated with Medical School		
No	336	87.73
Yes	46	12.01
Unknown	1	0.26

### 3.2 Physician opinions on insurance coverage for CPM

**Figure 1:**
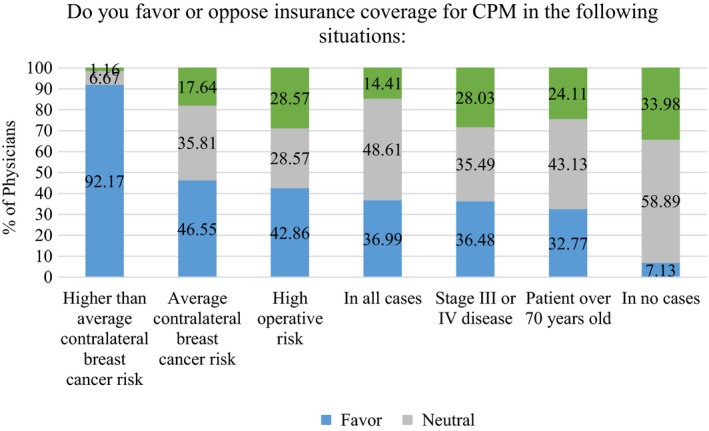
Physician opinion on insurance coverage for contralateral prophylactic mastectomy

Overall, 220 (22.8%) physicians stated that an insurance company denied coverage for CPM for at least one of their patients; this denial rate was highest in the South (n = 83, 26.8%) and West (n = 39, 27.9%) regions of the country and lowest in the Northeast region (n = 36, 15.6%). Figure 1 details physician opinions on insurance coverage for CPM. Most (n = 1036, 92.2%) felt that insurance should cover CPM for patients with higher than average CBC risk but for patients with average contralateral risk only 520 (46.6%) favored insurance coverage. There were statistically significant differences in opinions on insurance coverage by physician type. Within the physician subspecialties, 98 (34.0%) of medical oncologists, 86 (28.3%) of radiation oncologists, 167 (75.6%) of plastic surgeons, and 169 (55.6%) of surgeons favored insurance coverage for woman at average CBC risk (Appendix Table S1). The percentage of physicians favoring insurance coverage in all cases was 148 (63.8%) of plastic surgeons, only 57 (21.2%) (n = 57) of medical oncologists and 49 (17.5%) of radiation oncologists.

### 3.3 Physician opinions on whether CPM should be a quality measure

**Figure 2:**
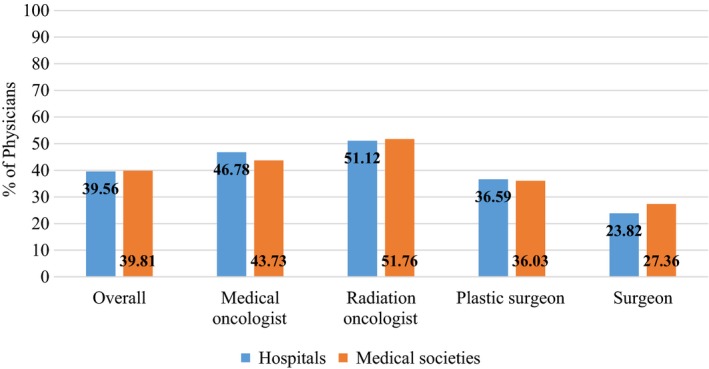
Proportion of physicians that felt contralateral prophylactic mastectomy should be a quality measure

Physicians were asked if physician specific CPM should be added as a quality measure by hospitals and medical societies (Figure 2). Approximately 35%‐40% of all physicians felt CPM should be a quality measure tracked by hospitals or medical societies. A significantly higher proportion of medical and radiation oncologists compared to plastic or breast surgeons felt physician specific rates of CPM should be added as a quality measure by hospitals and medical societies.

### 3.4 Physician opinions when CPM is strongly indicated vs discouraged

**Figure 3:**
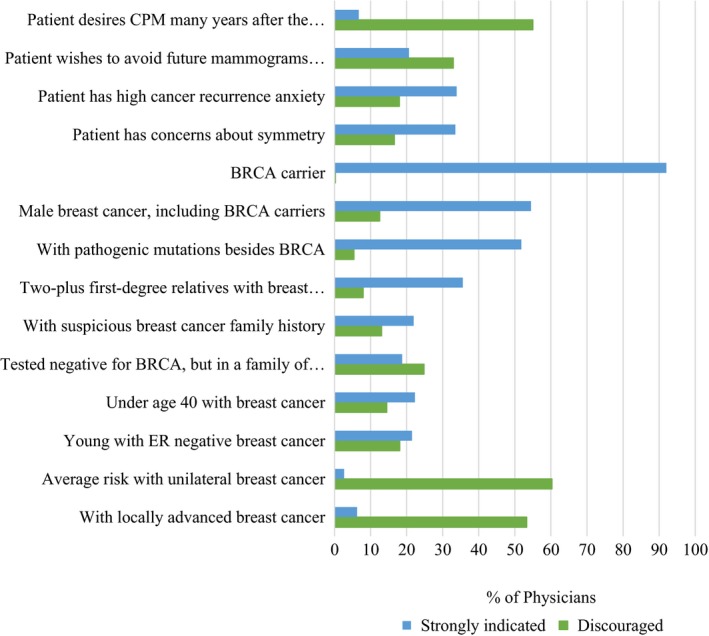
Proportion of physicians who felt that contralateral prophylactic mastectomy was strongly indicated or discouraged

The three main indications for CPM were (a) *BRCA* carriers, (b) male breast cancer, or (c) presence of germline mutations. The top 3 reasons to discourage CPM were (a) patient has average CBC risk, (b) locally advanced breast cancer, and (c) desire to have CPM many years after the original surgery (Figure 3). Responses to indications were stratified by physician type (Appendix Figure S1), demonstrating that plastic surgeons were significantly more likely to feel that CPM was strongly indicated in most scenarios compared with other physician subspecialties.

### 3.5 Independent physician factors associated with recommendation for CPM

Multivariable analysis of physician and practice factors was performed to determine independent factors associated with surgeon recommendation for CPM. Models were run for the following clinical scenarios: patient wishes to avoid future imaging and biopsies, patient has cancer recurrence anxiety, patient has concerns about symmetry, patient has family history, patient young age, patient is at average CBC risk, and patient has a locally advanced breast cancer. The only independent physician factor associated with recommending CPM for all the aforementioned scenarios was physician type. Compared to other physician types, plastic surgeons were significantly more likely to feel CPM was strongly indicated for all the aforementioned reasons (Table 2). When the model was run including only surgeons and plastics surgeons, the only physician factor found to be significant on multi‐variable analysis was physician gender (Appendix Table S2), such that female surgeon physicians were less likely to feel that CPM was strongly indicated for all the aforementioned reasons than male surgeons.

**Table 2:** Independent physician factors associated with physician opinion on indications for contralateral prophylactic mastectomy

Clinical scenario	Likelihood to recommend CPM#	*P* value
Patient wishes to avoid future mammograms or biopsies	**Plastic surgeon OR 5.6 (95%CI 3.5‐8.8) (Ref: medical oncologist)**	<.001
Patient has high cancer recurrence anxiety	**Plastic surgeon OR 5.6 (95%CI 3.8‐8.4) (Ref: medical oncologist)**	<.001
Patient has concerns about symmetry	**Plastic surgeon OR 3.2 (95%CI 2.2‐4.7) (Ref: medical oncologist)**	<.001
	Surgeon OR 1.5 (95%CI 1.1‐2.2) (Ref: medical oncologist)	.022
	10‐15 years in practice OR 1.7 (95%CI: 1.1‐2.6) (Ref: <5 years in practice)	.026
Patient has two‐plus first‐degree relatives with breast cancer	**Plastic surgeon OR 3.6 (95%CI 2.4‐5.3) (Ref: medical oncologist)**	<.001
	Surgeon OR 0.6 (95%CI 0.4‐0.9) (Ref: medical oncologist)	.020
	Radiation Oncologist OR 0.5 (95%CI: 0.4‐0.8) (Ref: medical oncologist)	.002
	
Suspicious breast cancer family history	**Plastic surgeon OR 4.7 (95%CI 3.0‐7.3) (Ref: medical oncologist)**	<.001
	5‐9 years in practice OR 1.9 (95%CI 1.1‐3.3) (Ref: <5 years in practice)	.021
	West facility location OR 1.7 (95%CI 1.0‐2.9) (Ref: Northeast region)	.043
Under age 40 years old with breast cancer	**Plastic surgeon OR 5.3 (95%CI 3.4‐8.4) (Ref: medical oncologist)**	<.001
	Surgeon OR 2.0 (95%CI 1.3‐3.1) (Ref: medical oncologist)	.003
	10‐15 years in practice OR 2.0 (95%CI 1.1‐3.4) (Ref: <5 years in practice)	.017
Young with ER negative breast cancer	**Plastic surgeon OR 7.9 (95%CI 4.9‐12.7) (Ref: medical oncologist)**	<.001
	10‐15 years in practice OR 1.9 (95%CI 1.1‐3.3) (ref: <5 years in practice)	.033
Average risk with unilateral breast cancer	**Plastic surgeon OR 8.3 (95%CI 2.4‐29.1) (Ref: medical oncologist)**	<.001
Locally advanced breast cancer	**Plastic surgeon OR 3.6 (95%CI 1.9‐6.9) (Ref: medical oncologist)**	<.001
	Surgeon OR 0.4 (95%CI 0.1‐0.9) (Ref: medical oncologist)	.035

Abbreviation: CPM, contralateral prophylactic mastectomy.

^#^ Multivariable model adjusting for physician type, gender, years in practice, number of patients seen per week, facility location, and practicing at a facility with a medical school affiliation.

## 4 DISCUSSION

Our study is the first large‐scale assessment of physician opinions on controversial issues regarding CPM. Physicians of different subspecialties were queried on their opinions on insurance coverage, CPM as a quality measure and indications to recommend CPM. Over 1200 physicians from 383 NAPBC‐accredited breast centers across the country participated in the study with an approximately 60% response rate from these centers. There was consensus that CPM is indicated for *BRCA* carriers and other pathogenic mutations but not indicated for those at average CBC risk. There was consensus that insurance should coverage CPM for BRCA carriers and approximately half felt insurance should cover CPM for women at average CBC risk. Likewise, less than half of physicians endorse CPM as a quality measure for hospitals or medical societies. These findings can guide discussions on CPM, particularly when to recommend or not recommend CPM and whether it is worthwhile to pursue CPM rates as a quality project at the individual hospital or center.

Many of our findings are consistent with other studies that have examined surgeon opinion on indications for CPM,^4^, ^12^, ^13^ however, a notable finding from our study was the difference in CPM recommendations among the physician subspecialties. Plastic surgeons were significantly more likely to recommend CPM than other physician specialties for nearly every clinical scenario. In some cases they were almost eight times as likely to recommend CPM than other physician subspecialties. These findings are not surprising given that a recent survey of American Society of Plastic Surgeons showed that only 18% felt uncomfortable with a patient's choice to have CPM^5^ in contrast to a survey of breast surgeons that showed 50% were uncomfortable performing CPM.^4^ These findings are significant since the majority of patients who undergo CPM undergo reconstruction^14^ and therefore are consulting with plastic surgeons about the pros and cons of unilateral mastectomy with or without CPM. Our findings demonstrate the wide disparity in physician opinions for CPM and why any future interventions in CPM decision‐making require involvement of not only surgeons but plastic surgeons as well.

Another interesting finding from our study was physician opinions on CPM as a quality measure. Almost half of radiation and medical oncologists felt physician specific rates of CPM should be a quality measure for hospitals and medical societies but only a quarter of surgeons felt the same way. Although CPM could impact outcomes that are important quality measures such as operative complications or delays in care, it is not clear if this impact is large enough to warrant CPM as a quality measure. Much of the CPM choice is patient driven and instituting CPM as a quality measure could limit patient decision‐making autonomy. Further investigation is needed on this topic.

We also asked physician opinions on insurance coverage for CPM. Although contralateral mastectomy is guaranteed coverage by the federally mandated Women's Health and Cancer Rights Act (WHCRA) for symmetry reasons,^15^ just under 40% of physicians felt insurance should cover CPM in all cases. Most surprisingly, despite the WHCRA, approximately one fifth of physicians reported that insurance had denied coverage for CPM for at least one of their patients. It will be interesting to see if denials of CPM coverage continue to increase over time with future cost containment measures in our healthcare system.

Some noteworthy findings surfaced when we examined opinions among just plastics and breast surgeons. Female surgeons were anywhere from 30% to 50% less likely to recommend CPM than male surgeons despite their age, or other factors. Interestingly, other studies have shown that CPM rates among female surgeons are higher than male surgeons.^16^ These dissimilarities in findings may be related to differences in the era of treatment, as these earlier studies may not be reflecting the current attitudes about CPM, particularly of female surgeons relative to male surgeons. This finding may also stem from male surgeons not wanting to be stereotyped as paternalistic and wanting to respect a woman's autonomy in CPM choice. They may also be related to the training and education that female surgeons receive about CPM since female surgeons had higher rates of fellowship training than the male surgeons.

Our data here show that most physicians feel CPM is indicated for hereditary factors despite the lack of robust literature demonstrating a clear survival advantage for CPM. In contrast to other studies, we asked about other hereditary factors besides just *BRCA* carrier status. Physicians felt pretty strong that CPM is indicated for men with breast cancer, women with first degree relatives with breast cancer and for those women with other pathogenic mutations besides *BRCA*. Few studies have examined CBC risk for male patients or male *BRCA* carriers. CBC risk for other non‐*BRCA* mutations is still largely unknown although some studies have shown higher CBC risk for CHEK2^17^ and PALB2^18^ although not for ATM.^19^, ^20^ CBC risk for patients having a significant family history is greater than for those patients without a family history^21^ although differences are small. Nonetheless, the survival benefit of CPM for patients with higher than average CBC risk has not been well established. Studies in *BRCA* carriers that have shown survival benefit to CPM were retrospective, did not include modern adjuvant therapies for breast cancer and in many cases patients found out they carried a *BRCA* mutation status many years after the original breast cancer diagnosis.^22^, ^23^ Therefore, it remains largely unknown if CPM really impacts overall survival for patients with increased CBC risk, including *BRCA* carriers who are at the highest CBC risk.

Our study has limitations, in that it was a cross‐sectional survey of physicians and we were not able to determine whether physician recommendations correlated with the actual surgery performed. We conducted a limited set of interviews with physicians so it is possible there are indications for CPM that we have missed. These data represent physician opinions and are not evidence‐based recommendations. In an effort to protect privacy of our accredited centers, demographic characteristics of the centers were not correlated with physician responses. However, our large numbers of centers across the United States and the relatively high response rate suggest that our findings are likely to accurately represent physician opinions of CPM across breast centers around the country.

In conclusion, while physician responses for CPM indications seem to globally include *BRCA* carriers and male breast cancer, there was no universal consensus for other indications for CPM, nor for who should be eligible for insurance coverage. Furthermore, there was no agreement for whether CPM rates would be a worthy quality measure. These findings underscore the complex nature of the decision‐making process for CPM, and given the differences in responses by subspecialty, suggests that future investigations, interventions, and decisions for quality measures should include a multidisciplinary physician team approach.

## CONFLICT OF INTEREST

The authors have no conflicts to disclose.

## AUTHORS CONTRIBUTION

Yao, Bleicher, Connolly, and Kurtzman were involved in conceptualization. Yao, Bleicher, and Chang were involved in data curation. Yao, Bleicher, Moran, and Chang were involved in formal analysis. Yao was involved in funding acquisition. Yao, Bleicher, Chang, and Kurtzman were involved in investigation. Yao, Bleicher, and Moran were involved in writing and original draft. Yao, Bleicher, Moran, Connolly, Dietz, Sarantou, and Kurtzman were involved in writing, and review and editing. All authors contributed to this study according to the guidelines set forth by *Cancer Medicine.* All authors made substantial contributions to conception and design, analysis and interpretation of data, were involved in drafting and revising the manuscript, gave final approval of the version to be published and agreed to be accountable for all aspects of the work in ensuring that questions related to the accuracy or integrity of any part of the work are appropriately investigated and resolved.

## Data Availability

The data that support the findings of this study are available from the corresponding author upon reasonable request.

## References

[1] Choosing wisely: promoting conversations between patients and clinicians. http://www.choosingwisely.org. Published 2019. Accessed.

[2] Boughey JC , Attai DJ , Chen SL , et al. Contralateral prophylactic mastectomy consensus statement from the American Society of Breast Surgeons: additional considerations and a framework for shared decision making. Ann Surg Oncol. 2016;23(10):3106‐3111. 27469118 10.1245/s10434-016-5408-8 PMC4999472

[3] NCCN guidelines. https://www.nccn.org. Published 2019. Accessed.

[4] Bellavance E , Peppercorn J , Kronsberg S , et al. Surgeons' perspectives of contralateral prophylactic mastectomy. Ann Surg Oncol. 2016;23(9):2779‐2787. 27169770 10.1245/s10434-016-5253-9

[5] Lopez CD , Bluebond‐Langner R , Houssock CA , Slezak SS , Bellavance E . Plastic and reconstructive surgeons' knowledge and comfort of contralateral prophylactic mastectomy: a survey of the american society of plastic surgeons. Front Oncol. 2018;8:647. 30687634 10.3389/fonc.2018.00647 PMC6334534

[6] Miller ME , Czechura T , Martz B , et al. Operative risks associated with contralateral prophylactic mastectomy: a single institution experience. Ann Surg Oncol. 2013;20(13):4113‐4120. 23868655 10.1245/s10434-013-3108-1

[7] Osman F , Saleh F , Jackson TD , Corrigan MA , Cil T . Increased postoperative complications in bilateral mastectomy patients compared to unilateral mastectomy: an analysis of the NSQIP database. Ann Surg Oncol. 2013;20(10):3212‐3217. 23846780 10.1245/s10434-013-3116-1

[8] Crosby MA , Garvey PB , Selber JC , et al. Reconstructive outcomes in patients undergoing contralateral prophylactic mastectomy. Plast Reconstr Surg. 2011;128(5):1025‐1033. 22030485 10.1097/PRS.0b013e31822b6682

[9] Liederbach E , Sisco M , Wang C , et al. Wait times for breast surgical operations, 2003–2011: a report from the National Cancer Data Base. Ann Surg Oncol. 2015;22(3):899‐907. 25234018 10.1245/s10434-014-4086-7

[10] Bleicher RJ , Ruth K , Sigurdson ER , et al. Preoperative delays in the US Medicare population with breast cancer. J Clin Oncol. 2012;30(36):4485‐4492. 23169513 10.1200/JCO.2012.41.7972 PMC3518727

[11] Miller ME , Bleicher RJ , Kaufman CS , et al. Impact of breast center accreditation on compliance with breast quality performance measures at commission on cancer‐accredited centers. Ann Surg Oncol. 2019;26(5):1202‐1211. 30684159 10.1245/s10434-018-07108-7

[12] Katz SJ , Hawley ST , Hamilton AS , et al. Surgeon influence on variation in receipt of contralateral prophylactic mastectomy for women with breast cancer. JAMA Surgery. 2018;153(1):29‐36. 28903158 10.1001/jamasurg.2017.3415 PMC5833615

[13] Musiello T , Bornhammar E , Saunders C . Breast surgeons' perceptions and attitudes towards contralateral prophylactic mastectomy. ANZ J Surg. 2013;83(7–8):527‐532. 23043449 10.1111/j.1445-2197.2012.06209.x

[14] Rosenberg SM , Tracy MS , Meyer ME , et al. Perceptions, knowledge, and satisfaction with contralateral prophylactic mastectomy among young women with breast cancer: a cross‐sectional survey. Ann Intern Med. 2013;159(6):373‐381. 24042365 10.7326/0003-4819-159-6-201309170-00003 PMC3968260

[15] Womens Health and Cancer Right Act. https://www.cancer.org/treatment/finding-and-paying-for-treatment. Published. 2019;Accessed 10/2019:2019.

[16] Arrington AK , Jarosek SL , Virnig BA , Habermann EB , Tuttle TM . Patient and surgeon characteristics associated with increased use of contralateral prophylactic mastectomy in patients with breast cancer. Ann Surg Oncol. 2009;16(10):2697‐2704. 19653045 10.1245/s10434-009-0641-z

[17] Kriege M , Hollestelle A , Jager A , et al. Survival and contralateral breast cancer in CHEK2 1100delC breast cancer patients: impact of adjuvant chemotherapy. Br J Cancer. 1100delC;111(5):1004‐1013. 10.1038/bjc.2014.306 PMC4150261 24918820

[18] Tischkowitz M , Capanu M , Sabbaghian N , et al. Rare germline mutations in PALB2 and breast cancer risk: a population‐based study. Hum Mutat. 2012;33(4):674‐680. 22241545 10.1002/humu.22022 PMC3767757

[19] Bernstein JL , Group WSC , Concannon P . ATM, radiation, and the risk of second primary breast cancer. Int J Radiat Biol. 2017;93(10):1121‐1127. 28627265 10.1080/09553002.2017.1344363 PMC6113688

[20] Bernstein JL , Haile RW , Stovall M , et al. Radiation exposure, the ATM Gene, and contralateral breast cancer in the women's environmental cancer and radiation epidemiology study. J Natl Cancer Inst. 2010;102(7):475‐483. 20305132 10.1093/jnci/djq055 PMC2902825

[21] Reiner AS , Sisti J , John EM , et al. Breast cancer family history and contralateral breast cancer risk in young women: an update from the women's environmental cancer and radiation epidemiology study. J Clin Oncol. 2018;36(15):1513‐1520. 29620998 10.1200/JCO.2017.77.3424 PMC5959199

[22] Heemskerk‐Gerritsen BA , Rookus MA , Aalfs CM , et al. Improved overall survival after contralateral risk‐reducing mastectomy in BRCA1/2 mutation carriers with a history of unilateral breast cancer: a prospective analysis. Int J Cancer. 2015;136(3):668‐677. 24947112 10.1002/ijc.29032

[23] Evans DGR , Ingham SL , Baildam A , et al. Contralateral mastectomy improves survival in women with BRCA1/2‐associated breast cancer. Breast Cancer Res Treat. 2013;140(1):135‐142. 23784379 10.1007/s10549-013-2583-1

