# Supplementary material for: Differences in physician opinions about controversial issues surrounding contralateral prophylactic mastectomy (CPM): A survey of physicians from accredited breast centers in the United States
Source: Cancer Med. 2020 Mar 11;9(9):3088–96. doi: 10.1002/cam4.2914 (PMC7196050; doi:10.1002/cam4.2914)
Supplement: Supplementary file 1 [file CAM4-9-3088-s001.docx]

**APPENDIX:**

**Table I.** Physician Opinion on Insurance Coverage for Contralateral Prophylactic Mastectomy Stratified by Physician Type

|  | Overall (N=1186) | | Medical Oncologist (N=300) | | Radiation Oncologist (N=316) | | Plastic Surgeon (N=248) | | Surgeon (N=322) | |  |
| --- | --- | --- | --- | --- | --- | --- | --- | --- | --- | --- | --- |
|  | N | % | N | % | N | % | N | % | N | % | p-value |
| **Do you favor or oppose insurance coverage for CPM in the following situations:** |  |  |  |  |  |  |  |  |  |  |  |
| High operative risk |  |  |  |  |  |  |  |  |  |  |  |
| Favor | 385 | 42.86 | 66 | 23.00 | 57 | 18.75 | 135 | 61.64 | 127 | 42.19 | **<.0001** |
| Neutral | 445 | 28.57 | 118 | 41.11 | 148 | 48.68 | 64 | 29.22 | 115 | 38.21 |  |
| Oppose | 281 | 28.57 | 103 | 35.89 | 99 | 32.57 | 20 | 9.13 | 59 | 19.60 |  |
| Average contralateral breast cancer risk |  |  |  |  |  |  |  |  |  |  |  |
| Favor | 520 | 46.55 | 98 | 34.03 | 86 | 28.29 | 167 | 75.57 | 169 | 55.59 | **<.0001** |
| Neutral | 400 | 35.81 | 130 | 45.14 | 124 | 40.79 | 49 | 22.17 | 97 | 31.91 |  |
| Oppose | 197 | 17.64 | 60 | 20.83 | 94 | 30.92 | 5 | 2.26 | 38 | 12.50 |  |
| Higher than average contralateral breast cancer risk |  |  |  |  |  |  |  |  |  |  |  |
| Favor | 1036 | 92.17 | 267 | 92.07 | 271 | 87.99 | 214 | 96.40 | 284 | 93.42 | **0.0195** |
| Neutral | 75 | 6.67 | 21 | 7.24 | 30 | 9.74 | 7 | 3.15 | 17 | 5.59 |  |
| Oppose | 13 | 1.16 | 2 | 0.69 | 7 | 2.27 | 1 | 0.45 | 3 | 0.99 |  |
| Stage III or IV disease |  |  |  |  |  |  |  |  |  |  |  |
| Favor | 406 | 36.48 | 75 | 26.13 | 71 | 23.36 | 150 | 68.81 | 110 | 36.18 | **<.0001** |
| Neutral | 395 | 35.49 | 113 | 39.37 | 115 | 37.83 | 57 | 26.15 | 110 | 36.18 |  |
| Oppose | 312 | 28.03 | 99 | 34.49 | 118 | 38.82 | 11 | 5.05 | 84 | 27.63 |  |
| Patient over 70 years old |  |  |  |  |  |  |  |  |  |  |  |
| Favor | 367 | 32.77 | 65 | 22.49 | 64 | 20.92 | 123 | 55.66 | 115 | 37.83 | **<.0001** |
| Neutral | 483 | 43.13 | 140 | 48.44 | 132 | 43.14 | 82 | 37.10 | 129 | 42.43 |  |
| Oppose | 270 | 24.11 | 84 | 29.07 | 110 | 35.95 | 16 | 7.24 | 60 | 19.74 |  |
| In all cases |  |  |  |  |  |  |  |  |  |  |  |
| Favor | 398 | 36.99 | 57 | 21.19 | 49 | 17.50 | 148 | 63.79 | 144 | 48.81 | **<.0001** |
| Neutral | 523 | 48.61 | 163 | 60.59 | 169 | 60.36 | 68 | 29.31 | 123 | 41.69 |  |
| Oppose | 155 | 14.41 | 49 | 18.22 | 62 | 22.14 | 16 | 6.90 | 28 | 9.49 |  |
| In no cases |  |  |  |  |  |  |  |  |  |  |  |
| Favor | 63 | 7.13 | 14 | 5.71 | 8 | 3.23 | 21 | 13.64 | 20 | 8.47 | **<.0001** |
| Neutral | 520 | 58.89 | 158 | 64.49 | 177 | 71.37 | 60 | 38.96 | 125 | 52.97 |  |
| Oppose | 300 | 33.98 | 73 | 29.80 | 63 | 25.40 | 73 | 47.40 | 91 | 38.56 |  |

**Figure I.** Proportion of Physicians who Felt that Contralateral Prophylactic Mastectomy was Strongly Indicated Stratified by Physician Type

**Table II**. Independent Physician Factors Associated with Physician Opinion on Indications for Contralateral Prophylactic Mastectomy (Confined to plastic surgeon and surgeons only)

| **Clinical scenario** | **Likelihood to recommend CPM#** | **P value** |
| --- | --- | --- |
| Patient wishes to avoid future mammograms or biopsies | Female OR 0.6 (95%CI 0.4-0.9) (Ref: male)  >20 years in practice OR 0.4 (95%CI 0.2-0.7) (Ref: <5yrs in practice) | 0.012  0.004 |
| Patient has high cancer recurrence anxiety | Female OR 0.5 (95%CI 0.3-0.7) (Ref: male)  >50 pts/week OR 0.5 (95%CI 0.3-0.9) (Ref: <10 pts per week) | <0.001  0.026 |
| Patient has concerns about symmetry | Female OR 0.7 (95%CI 0.5-1.0) (Ref: male) | 0.0314 |
| Patient has two-plus first-degree relatives with breast cancer | Female OR 0.5 (95%CI 0.4-0.8) (Ref: male)  10-29 pts/week OR 0.6 (95%CI 0.4-1.0) (Ref: <10 pts per week)  >50 pts/week OR 0.3 (95%CI 0.2-0.6) (Ref: <10 pts per week) | 0.001  0.043  <0.001 |
| Suspicious breast cancer family history | Female OR 0.6 (95%CI 0.4-0.9) (Ref: male)  10-29 pts/week OR 0.6 (95%CI 0.3-1.0) (Ref: <10 pts per week) | 0.011  0.031 |
| Under age 40 years old with breast cancer | Female OR 0.5 (95%CI 0.4-0.8) (Ref: male) | 0.002 |
| Young with ER negative breast cancer | Female OR 0.6(95%CI 0.4-0.9) (Ref: male)  5-9 years in practice OR 2.1 (95%CI 1.0-4.3) (Ref: <5 yrs in practice)  10-15 years in practice OR 2.2 (95%CI 1.1-4.5) (Ref: <5 yrs in practice)  >50 patients/week OR 0.4 (95%CI 0.2-0.7) (<10 pts per week) | 0.015  0.038  0.028  0.001 |
| Average risk with unilateral breast cancer | Female OR 0.2 (95%CI 0.1-0.6) (Ref: male) | 0.003 |
| Locally advanced breast cancer | Female OR 0.5 (95%CI 0.3-1.0) (Ref: male)    >20 years in practice OR 0.2 (95%CI 0.1-0.6)  (Ref: <5 yrs in practice)  >50 patients/week OR 0.2 (95%CI 0.1-0.7) (Ref: <10 pts per week) | 0.050  0.004  0.014 |

#Multivariable model adjusting for physician gender, years in practice, number of patients seen per week, facility location and practicing at a facility with a medical school affiliation

CPM, contralateral prophylactic mastectomy; pts, patients; yrs, years.
