# Supplementary material for: Differences in physician opinions about controversial issues surrounding contralateral prophylactic mastectomy (CPM): A survey of physicians from accredited breast centers in the United States
Source: Cancer Med. 2020 Mar 11;9(9):3088–96. doi: 10.1002/cam4.2914 (PMC7196050; doi:10.1002/cam4.2914)
Supplement: Supplementary file 2 [file CAM4-9-3088-s002.pdf]

CPM Decision Making
(Contralateral Prophylactic Mastectomy)

DCIS Management
(Ductal Carcinoma In Situ)

AND

National Accreditation Program for Breast Centers

A Survey of NAPBC Physicians
May 2017

Please note: Your participation in this survey is voluntary – you may skip questions you do not wish to answer. By completing and returning the survey, you provide your permission for us to use the data. Your response is confidential, but the Survey Lab does use completion rate at the center level to guide reminder effort. This means the Survey Lab will track the total completes by center, but *not* by individual, and will *not* enter the names of centers with the survey answers. Center names will *not* be retained beyond the data collection phase, nor shared with Dr. Yao or other study analysts or sponsors. Neither your name nor the name of your center will be part of the data set used for analysis.

Please return survey to:

The University of Chicago Survey Lab
6030 S. Ellis Ave. Rm 152
Chicago IL 60637

A postage-paid envelope is provided with the survey for this purpose.

SECTION A. Contralateral Prophylactic Mastectomy (CPM)

First we ask for your feelings and opinions about CPM (contralateral prophylactic mastectomy).

A1. Do you favor or oppose insurance coverage for CPM in the following situations?

	Favor Coverage	Neutral	Oppose Coverage
A. High operative risk.	①	②	③
B. Average contralateral breast cancer risk.	①	②	③
C. Higher than average contralateral breast cancer risk.	①	②	③
D. Stage III or IV disease.	①	②	③
E. Patient over 70 years old.	①	②	③
F. In all cases.	①	②	③
G. In no cases.	①	②	③

A2. Do you think *overall* rates of CPM should be added as a quality measure by ...

	Definitely yes	Probably yes	Probably not	Definitely not	No Opinion
A. Hospitals.	①	②	③	④	☐-2
B. Medical societies.	①	②	③	④	☐-2
C. Insurance companies.	①	②	③	④	☐-2

A3. Do you think *physician-specific* rates of CPM should be added as a quality measure by ...

	Definitely yes	Probably yes	Probably not	Definitely not	No Opinion
A. Hospitals.	①	②	③	④	☐-2
B. Medical societies.	①	②	③	④	☐-2
C. Insurance companies.	①	②	③	④	☐-2

A4. Has an insurance company ever denied coverage for CPM in one of your cases?

① Yes

② No

Next are some knowledge questions about CPM. Please make your best guess without looking up information.

A5. What is your best guess of the ten and twenty year risks of contralateral breast cancer in patients with ...

IDC and no additional risk factors	IDC and a first-degree relative with breast cancer	IDC and a BRCA gene mutation
A. 10-year contralateral risk ______%	C. 10-year contralateral risk ______%	E. 10-year contralateral risk ______%
B. 20-year contralateral risk ______%	D. 20-year contralateral risk ______%	F. 20-year contralateral risk ______%

A6. For disease at the same stage, how do local recurrence risks compare across tumor types for lumpectomy or mastectomy?

	Lumpectomy carries higher local recurrence risk	Same local recurrence risk for lumpectomy and mastectomy	Mastectomy carries higher local recurrence risk	Unsure
A. Triple negative.	①	②	③	☐-2
B. HER2neu positive.	①	②	③	☐-2
C. ER positive.	①	②	③	☐-2

Now are questions about when you believe CPM is indicated, should be discouraged, or neither.

A7. In these situations, would you say ...

	CPM is strongly indicated	Neither	CPM should be discouraged	No idea
A. Patient desires CPM many years after the original breast surgery.	①	②	③	☐-2
B. Patient wishes to avoid future mammograms or biopsies.	①	②	③	☐-2
C. Patient has high cancer recurrence anxiety.	①	②	③	☐-2
D. Patient has concerns about symmetry.	①	②	③	☐-2

A8. For these patients, would you say ...

	CPM is strongly indicated	Neither	CPM should be discouraged	No idea
A. Under age 40 with breast cancer.	①	②	③	☐-2
B. With suspicious breast cancer family history.	①	②	③	☐-2
C. BRCA carrier.	①	②	③	☐-2
D. Tested negative for BRCA, but in a family of BRCA positive carriers.	①	②	③	☐-4
E. Two-plus first-degree relatives with breast cancer.	①	②	③	☐-2
F. Male breast cancer, including BRCA carriers.	①	②	③	☐-2
G. Young with ER negative breast cancer.	①	②	③	☐-2
H. With pathogenic mutations besides BRCA.	①	②	③	☐-2
I. Average risk with unilateral breast cancer.	①	②	③	☐-2
J. With locally advanced breast cancer.	①	②	③	☐-2
